# Ezetimibe, Niemann-Pick C1 like 1 inhibitor, modulates hepatic phospholipid metabolism to alleviate fat accumulation

**DOI:** 10.3389/fphar.2024.1406493

**Published:** 2024-06-17

**Authors:** Hyekyung Yang, Dong Ho Suh, Eun Sung Jung, Yoonjin Lee, Kwang-Hyeon Liu, In-Gu Do, Choong Hwan Lee, Cheol-Young Park

**Affiliations:** ^1^ Medical Research Institute, Kangbuk Samsung Hospital, Sungkyunkwan University School of Medicine, Seoul, Republic of Korea; ^2^ Department of Bioscience and Biotechnology, Konkuk University, Seoul, Republic of Korea; ^3^ College of Pharmacy and Research Institute of Pharmaceutical Sciences, Kyungpook National University, Daegu, Republic of Korea; ^4^ Department of Pathology, Kangbuk Samsung Hospital, Sungkyunkwan University School of Medicine, Seoul, Republic of Korea; ^5^ Division of Endocrinology and Metabolism, Department of Internal Medicine, Kangbuk Samsung Hospital, Sungkyunkwan University School of Medicine, Seoul, Republic of Korea

**Keywords:** ezetimibe, hepatic steatosis, phospholipids, metabolomics, lipidomics

## Abstract

**Background:**

Ezetimibe, which lowers cholesterol by blocking the intestinal cholesterol transporter Niemann-Pick C1 like 1, is reported to reduce hepatic steatosis in humans and animals. Here, we demonstrate the changes in hepatic metabolites and lipids and explain the underlying mechanism of ezetimibe in hepatic steatosis.

**Methods:**

We fed Otsuka Long-Evans Tokushima Fatty (OLETF) rats a high-fat diet (60 kcal % fat) with or vehicle (control) or ezetimibe (10 mg kg^-1^) via stomach gavage for 12 weeks and performed comprehensive metabolomic and lipidomic profiling of liver tissue. We used rat liver tissues, HepG2 hepatoma cell lines, and siRNA to explore the underlying mechanism.

**Results:**

In OLETF rats on a high-fat diet, ezetimibe showed improvements in metabolic parameters and reduction in hepatic fat accumulation. The comprehensive metabolomic and lipidomic profiling revealed significant changes in phospholipids, particularly phosphatidylcholines (PC), and alterations in the fatty acyl-chain composition in hepatic PCs. Further analyses involving gene expression and triglyceride assessments in rat liver tissues, HepG2 hepatoma cell lines, and siRNA experiments unveiled that ezetimibe’s mechanism involves the upregulation of key phospholipid biosynthesis genes, CTP:phosphocholine cytidylyltransferase alpha and phosphatidylethanolamine N-methyl-transferase, and the phospholipid remodeling gene lysophosphatidylcholine acyltransferase 3.

**Conclusion:**

This study demonstrate that ezetimibe improves metabolic parameters and reduces hepatic fat accumulation by influencing the composition and levels of phospholipids, specifically phosphatidylcholines, and by upregulating genes related to phospholipid biosynthesis and remodeling. These findings provide valuable insights into the molecular pathways through which ezetimibe mitigates hepatic fat accumulation, emphasizing the role of phospholipid metabolism.

## 1 Introduction

Non-alcoholic fatty liver disease (NAFLD) is characterized by a massive accumulation of fat in the liver and represents a continuum of liver abnormalities from simple steatosis to progressive non-alcoholic steatohepatitis (NASH), which involves inflammation and fibrosis (Friedman et al., 2018). NAFLD is strongly associated with obesity, insulin resistance, hypertension, and dyslipidemia and is related to an increase in cardiovascular risk fibrosis (Rinella and Sanyal, 2016; Friedman et al., 2018). Despite the growing prevalence and incidence of NAFLD worldwide, no agent has been approved for the treatment of NAFLD (Rinella and Sanyal, 2016; Friedman et al., 2018).

Ezetimibe is a cholesterol absorption inhibitor that functions by binding Niemann-Pick C1 like 1 (NPC1L1) protein on enterocytes, decreasing the delivery of cholesterol to the liver (Garcia-Calvo et al., 2005). Ezetimibe treatment alone or in combination with statins has been shown to significantly reduce low-density lipoprotein cholesterol (LDL-C) and total cholesterol (TC) in hypercholesterolemia patients (Gagné et al., 2002; Ballantyne et al., 2003). In addition to its effect on hypercholesterolemia, ezetimibe can prevent hepatic steatosis in animal models and humans (Deushi et al., 2007; Yoneda et al., 2010; Takeshita et al., 2014; Chang et al., 2015b). However, there is a still lack of research on the underlying hepatic mechanisms related to ezetimibe treatment.

Metabolomic and lipidomics approaches have been used to explain the therapeutic mechanisms of NAFLD in various biofluids and tissues (Dumas et al., 2014). Some studies have demonstrated that metabolomics can provide information on the development of NAFLD in relation to the health of the liver, such as dysregulation of bile acid and phospholipid homeostasis (Beyoğlu and Idle, 2013), and the mechanisms of therapeutic agents and medicines (Terashima et al., 2014; Yang et al., 2018). Thus, metabolomics can explain the global physiological status in biochemical pathways and provide potential biomarkers, either by targeted key metabolite or global metabolite profiling, for use in diagnostics, disease development, drug discovery, and the treatment mechanisms of medicine (Wishart, 2016).

In this study, we investigated the effect of ezetimibe on liver histology and function in a rat model of obesity and type 2 diabetes (T2D). We performed comprehensive metabolomic and lipidomic profiling of liver tissue with various statistical analyses to explore the hepatic mechanism related to the therapeutic efficacy of ezetimibe. Our results provide insight into how ezetimibe regulates lipid metabolism, particularly the synthesis and remodeling of phospholipids, and ameliorates hepatic fat accumulation.

## 2 Materials and methods

### 2.1 Materials

Ezetimibe was obtained as a gift from Merck and CO. (Rahway, NJ, United States). Acetonitrile, methanol, chloroform, and water were obtained from Fisher Scientific (Pittsburgh, PA, United States). Formic acid, pyridine, methoxyamine-hydrochloride, N-methyl-N-(trimethylsilyl)-trifluoroacetamide, and standard compounds were purchased from Sigma-Aldrich (St. Louis, MO, United States). Scrambled small interference (si) RNA (AccuTarget™ Negative Control siRNA, SN-1003), human CCTα siRNA (siCCTα: sense 5′-CUC​CAA​CAC​AGA​GGA​CAG​A-3′; antisense 5′-UCU​GUC​CUC​UGU​GUU​GGA​G-3′) and human LPCAT3 siRNA (siLPCAT3: sense 5′-CUA​CUU​UGA​CGG​AGG​GAA-3′; antisense 5′-UUU​CCC​UCC​GUC​AAA​GUA​G-3′) were purchased from Bioneer (Daejeon, Rep. of Korea). Human PEMT siRNA (siPEMT: sense 5′-CCU​ACA​UAG​UGG​CUC​UCC​U-3′; antisense 5′-AGG​AGA​GCC​ACU​AUG​UAG​G-3′) was purchased from Sigma-Aldrich. All chemicals and solvents used in this study were of analytical reagent grade. Freshly distilled water was used in all experiments.

### 2.2 Animal experiments

#### 2.2.1 Animals

Animal studies were performed in compliance with the Animal Research: Reporting of *In Vivo* Experiments (ARRIVE) guidelines (Kilkenny et al., 2010). All animal experiments were approved by the Ethics Committee for Animal Experiments of Kangbuk Samsung Hospital, Sungkyunkwan University School of Medicine, and performed in accordance with the criteria of the “Guide for the Care and Use of Laboratory Animals” published by the United States National Institutes of Health (National Research Council Institute for Laboratory Animal, 1996). Male Otsuka Long-Evans Tokushima Fatty (OLETF) rats (150–200 g, 4 weeks old) were purchased from Otsuka Pharmaceutical (Tokushima, Japan). Rats were housed in individual cages with heated wood chip litter as bedding material in a pathogen-free environment and maintained in a temperature- and humidity-controlled room (24°C ± 2°C and 60% humidity) with a 12 h light/dark cycle; animals were fed standard irradiated rodent chow (11% kcal fat; LabDiet, St. Louis, MO, United States) and water *ad libitum*. Body weight and food intake were measured every week.

#### 2.2.2 HFD feed and ezetimibe treatment

To investigate the metabolic effects of ezetimibe on hepatic steatosis, we fed the animals a high-fat diet (60% kcal fat, 24% kcal carbohydrates, and 16% kcal protein, Research Diets D12492, New Brunswick, NJ, United States; HFD) at the age of 7 weeks. Rats were randomly divided into two groups: (1) control group with vehicle only (*n* = 8); and (2) ezetimibe group (10 mg⋅kg^−1^ per day, *n* = 8). Ezetimibe was dissolved in 0.5% sodium carboxymethyl cellulose and orally administered daily for 12 weeks. At the end of the experiment, rats were fasted overnight and anesthetized with isoflurane. Blood was collected from the abdominal aorta, and tissues were immediately dissected, weighed, and stored at −80°C until further analysis.

#### 2.2.3 Measurement of glucose tolerance

The glucose tolerance test (GTT) was carried out after an 18 h fast. Rats were orally administrated glucose solution (2 g⋅kg^-1^ body weight) and blood glucose levels were measured at baseline and 15, 30, 60, 90, and 120 min after injection using the automated glucocard X-Meter (Arkray, Kyoto, Japan). The area under the curve (AUC) was calculated.

#### 2.2.4 Measurement of metabolic parameters

Plasma insulin was analyzed by enzymatic assay (Crystal Chem, Downers Grove, IL, United States). Blood and liver triglyceride (TG) levels were also measured by enzymatic assay (ETGA-200, BioAssay Systems, Hayward, CA). Commercial kits were used for the measurement of free fatty acid (FFA; EFFA-100, BioAsssay Systems), total cholesterol (TC; STA-384, Cell Biolabs, Inc., Ann Arbor, MI, United States), LDL/VLDL (ab65390, Abcam, Cambridge, United Kingdom), phosphatidylcholine (PC; ab83377, Abcam), phosphatidylethanolamine (PE; ab241005, Abcam), and lysophosphatidylcholine (lysoPC; ab273332 Abcam), and activities of alanine aminotransferase (ALT; Sigma-Aldrich) and aspartate aminotransferase (AST; Sigma-Aldrich levels were analyzed using the commercial kits For hepatic TG quantification, lipid was extracted as described in a previous study (Chang et al., 2018). For hepatic FFA and TC analysis, liver lipids were extracted according to the modified Folch method (Folch et al., 1957; Alfaradhi et al., 2014). Liver metabolic parameters were normalized to liver weight.

#### 2.2.5 Histological analysis and NAFLD activity score

Dissected liver tissues were fixed in 10% formalin buffer overnight. The tissues were then embedded in paraffin, sliced into 4-μm-thick sections, and stained with hematoxylin and eosin (H&E). Digital images were captured with an Olympus BX51 light microscope (×100 magnification, Tokyo, Japan). A pathologist blinded to the experimental conditions evaluated the NAFLD activity score (NAS). Three features of NAFLD (steatosis, lobular inflammation, and ballooning) were scored as described previously (Chang et al., 2015b). NAS was calculated as the sum of scores for steatosis (0–3), ballooning (0–2), and inflammation (0–3). The diameter of lipid droplets (LDs) was measured using ImageJ software (ver. 1.52a; NIH, Bethesda, MD, United States) as described previously (Sasaki et al., 2020).

#### 2.2.6 Measurement of enzyme activity

Rat liver tissues were homogenized in cold phosphate-buffered saline (PBS) at pH 7.4 and centrifuged at 10,000 × *g* for 15 min at 4°C and the supernatants were collected. Enzyme activities of PLA_2_ and PC-PLC were assayed using the commercial assay kits according to the manufacturer’s instructions (EnzChek, Invitrogen).

### 2.3 Cell culture

#### 2.3.1 Cell culture and treatment

Human hepatocellular carcinoma HepG2 cells were obtained from American Type Culture Collection (Manassas, VA, United States) and cultured in Dulbecco’s modified Eagle’s medium (DMEM, Thermo Fisher Scientific, Rockford, IL, United States) supplemented with 10% v/v fetal bovine serum (FBS) and 100 units/mL antibiotic–antimycotic (Thermo Fisher Scientific). The ezetimibe stock solution was prepared in dimethylsulfoxide (DMSO; Sigma-Aldrich). Oleic acid (OA) (Sigma-Aldrich) was used to induce LD accumulation in HepG2 cells (Cui et al., 2010). The cells were treated with 50 μM ezetimibe for 1 h prior to incubation with 2 mM OA for 18 h. Cells were then harvested for either mRNA extraction or intracellular TG analysis.

#### 2.3.2 siRNA transfection

siRNAs (200 pmol) targeting human CCTα, LPCAT3, and PEMT and scrambled siRNA were transfected using Lipofectamine RNAiMAX (Invitrogen, Carlsbad, CA, United States) according to the manufacturer’s protocol. After 2 days, cells were treated with 50 μM ezetimibe for 1 h prior to incubation with 2 mM OA for 18 h. Cells were then harvested for subsequent analysis.

### 2.4 Gene expression analysis

Total RNA was extracted from tissues using TRIzol Reagent (Invitrogen, Carlsbad, CA, United States) and reverse-transcribed to cDNA using a High-Capacity RNA-to-cDNA kit (Applied Biosystems, Foster City, CA, United States) according to the manufacturer’s instructions. Gene expression levels were analyzed by real-time polymerase chain reaction (PCR) using a LightCycler 480 System (Roche Diagnostics, Indianapolis, IN, United States) based on SYBR Green fluorescence signals. The PCR parameters were as follows: pre-denaturation at 95°C for 10 min, followed by 40 cycles of denaturation at 95°C for 10 s, annealing at 56°C for 10 s, and extension at 72°C for 20 s. The primer sequences are listed in Supplementary Table S1. The mRNA expression of each target was normalized to β-actin mRNA as an internal control and expressed as fold change relative to the control group.

### 2.5 Statistical analysis

Data are represented as mean ± standard error of mean (SEM) unless specified otherwise. Unless mentioned, significant differences among experimental groups (*p-value*) were evaluated by Student’s t-test (paired, two-sided). *p* < 0.05 was considered significant. All authors had access to the study data and had reviewed and approved the final manuscript.

### 2.6 Metabolomic and lipidomic analysis

#### 2.6.1 Hepatic metabolite extraction for metabolomic and lipidomic analyses

Metabolites were extracted from the liver tissue (100 mg) of each mouse with 50% cold-methanol (1 mL, methanol:water, v/v) and 10 μL of internal standard (2-chlorophenylalanine, 0.5 mg·mL^−1^) using an MM400 mixer mill (Retsch^®^, Haan, Germany) at a frequency of 30 s^−1^ for 10 min with zirconium beads. The suspension was cold centrifuged at 12,578 *g* for 10 min. The supernatant was filtered through a 0.2 μm PTFE filter and evaporated using a speed vacuum concentrator (Modulspin 31; BioTron, Inc., Korea). The final concentration of each sample was prepared as 5 mg/mL using methanol.

For lipidomic analysis, lipids were extracted from liver tissue (100 mg) with a solvent mixture (1 mL, chloroform/methanol, 1:2, *v/v*) using a mixer mill at a frequency of 30 s^−1^ for 2 min. After a 1 h incubation at RT, 300 μL of chloroform and 450 μL of water were added; the sample was vortexed and cold centrifuged (1,000 *g* and 10 min). The lower phase was transferred to a new tube, and the upper phase was re-extracted using 600 μL of chloroform. Pooled chloroform extracts were evaporated using a speed vacuum concentrator. Each dried extract was reconstituted in 100 μL of mixture solvent (methanol:chloroform, 9:1, *v/v*) and diluted 50-fold with a solvent mixture containing 7.5 mM ammonium acetate.

#### 2.6.2 Metabolomic and lipidomic analysis

Gas chromatography–time of flight-mass spectrometry (GC-TOF-MS), ultra-performance liquid chromatography-quadrupole-MS (UPLC-Q-TOF-MS), nanomate-LTQ-MS, and raw data processing were performed as previously described (Yang et al., 2018). Briefly, 1 μL of derivatized samples was injected in the splitless mode in GC-TOF-MS, 5 μL of the sample was analyzed by UPLC-Q-TOF-MS, and 10 μL of each sample was injected into the Thermo LTQ XL ion trap MS. The fitness and prediction value of orthogonal partial least squares discriminant analysis (OPLS-DA) models was evaluated by R2X, R2Y, Q2, and *p-value*. To obtain reliable MS data, a quality control sample was loaded at blocks of 10 runs during the MS analysis, and all metabolomic and lipidomic data were normalized using an internal standard.

#### 2.6.3 Statistical analysis for metabolomic and lipidomic analyses

Significant differences among experimental groups (*p* values) and correlation coefficients were evaluated using PASW Statistics 18 software (SPSS Inc., Chicago, IL, United States). The heatmap and correlation network were constructed by MeV software (http://www.tm4.org/) and Cytoscape software (version 3.4.0, http://www.cytoscape.org), respectively. The diagnostic accuracy of the model was performed using the area under the receiver operating characteristic (ROC) curve (AUC). The ROC curves and logistic regression analyses were generated using MedCalc software (version 14.8.1, MedCalc Software Ltd., Ostend, Belgium). Multivariate statistical analysis including principal component analysis (PCA) and OPLS-DA was conducted using SIMCA P+ (version 12.0, Umetrics, UMEÅ, Sweden). Significantly different hepatic metabolites were selected based on variable importance projection (VIP) values, and significance was tested by analysis of variance (*t*-test, PASW statistics 18 software) between experimental groups.

## 3 Results

### 3.1 Ezetimibe significantly improves metabolic parameters and liver function

**FIGURE 1:**
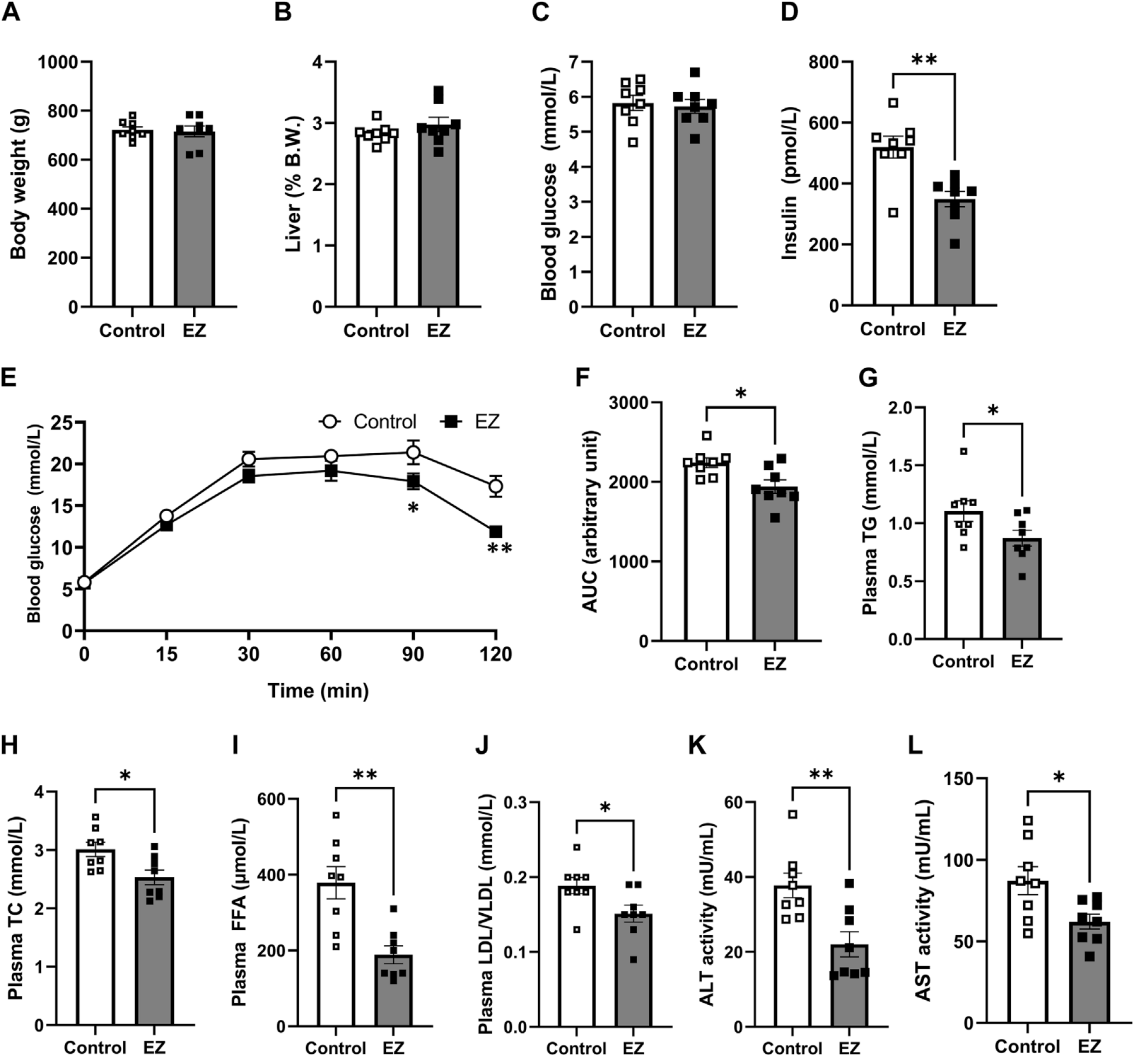
Effects of ezetimibe on metabolic parameters. **(A)** Body weight. **(B)** Liver weight shown as a percentage of body weight. **(C)** Blood glucose level. **(D)** Plasma insulin level. **(E, F)** Effect of ezetimibe on oral glucose tolerance. Plasma levels of **(G)** total triglyceride (TG), **(H)** total cholesterol (TC), **(I)** free fatty acids (FFA), and **(J)** LDL/VLDL. Activities of **(K)** ALT and **(L)** AST. Data are expressed as the mean ± SEM (*n* = 8 for each group). *p < 0.05, **p < 0.01 vs. control, by Student’s t-test. Abbreviations: ALT: alanine aminotransferase; AST: aspartate aminotransferase; CONT: control; EZ: ezetimibe; LDL: low-density lipoprotein cholesterol; VLDL: very low-density lipoprotein cholesterol. SEM: standard error of mean.

To investigate the effects of ezetimibe on metabolic regulation, OLETF rats were fed a high-fat diet for 12 weeks. At 3 months on a high-fat diet, changes in body weight and liver weight were similar between the control and the ezetimibe-treated groups (Figures 1A, B). There was no significant difference in the level of plasma glucose between groups (Figure 1C). However, the ezetimibe-treated group showed a decrease in the plasma insulin level, along with a significant improvement in glucose tolerance compared with control rats (Figures 1D–F). Lipid profiles in plasma, including TG, TC, FFAs, and LDL/VLDL-C, were improved with ezetimibe treatment ( Figures 1G–J ). The activities of liver enzymes (ALT and AST) were 43% (*p* < 0.05) and 32% (*p* < 0.05) lower, respectively, in the ezetimibe-treated group than those in the control group (Figures 1K, L), indicative of an improvement in overall liver function.

### 3.2 Ezetimibe effectively ameliorates hepatic fat accumulation

**FIGURE 2:**
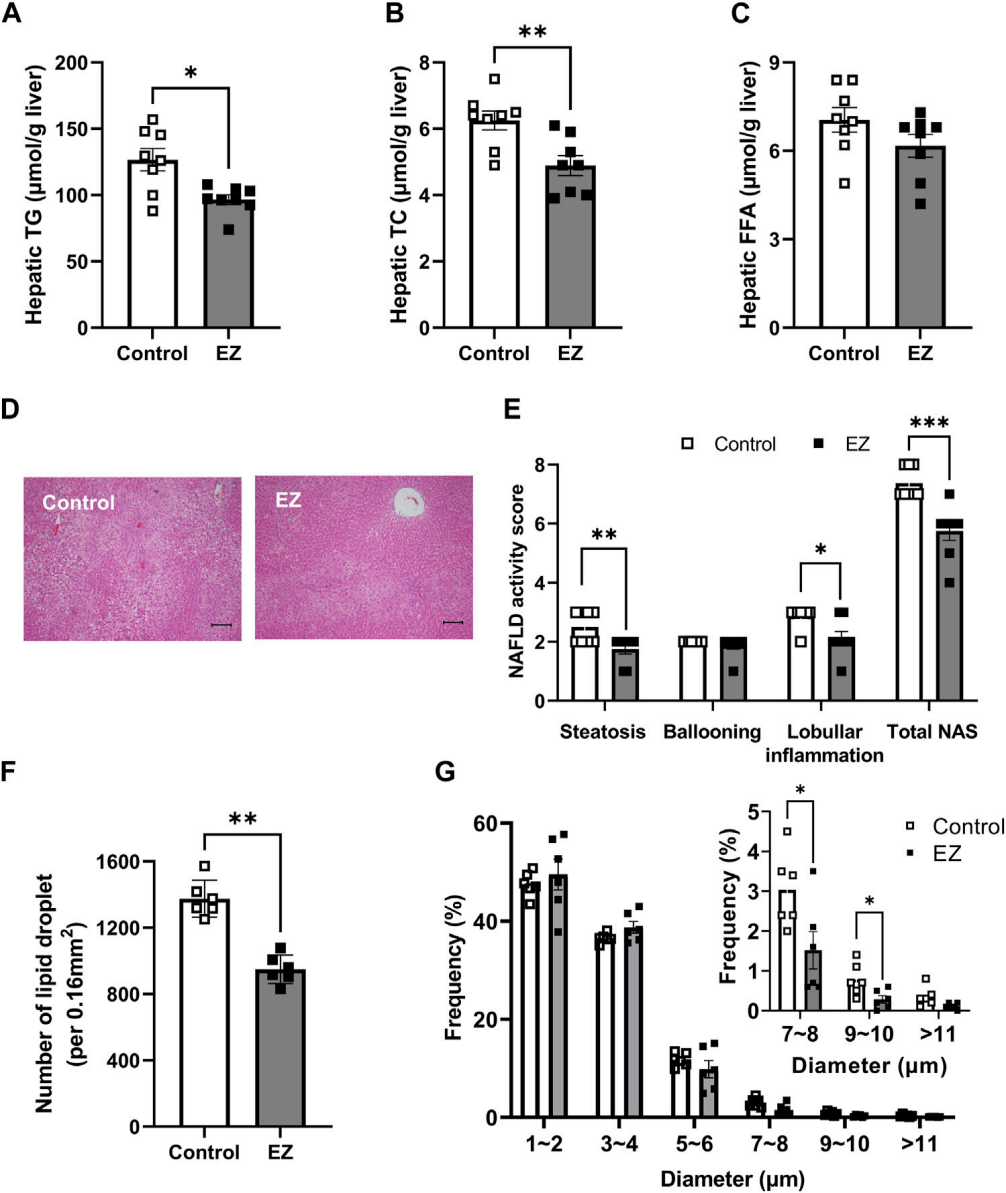
Effects of ezetimibe on hepatic fat accumulation. Hepatic levels of **(A)** total triglyceride (TG), **(B)** total cholesterol (TC), and **(C)** free fatty acids (FFA) (*n* = 8 for each group). **(D)** Representative H&E-stained liver sections (scale bar, 100 µm). Digital images were captured with an Olympus BX51 light microscope (Tokyo, Japan). **(E)** Evaluation of NAFLD activity score (NAS). A pathologist blinded to the experimental conditions evaluated the NAFLD activity score (NAS). NAS was calculated as the sum of scores for steatosis (0–3), ballooning (0–2), and inflammation (0–3) (*n* = 8 for each group). **(F, G)** Quantitative analysis of the average of the lipid droplet number **(F)** and distribution of lipid droplets in diameter **(G)** (*n* = 6 for each group). Each data point is an average of 3 different fields of view per rat. The diameter of lipid droplets (LDs) was measured using ImageJ software. Data are expressed as the mean ± SEM. *p < 0.05, **p < 0.01, ***p < 0.001 vs. control, by Student’s t-test. Abbreviations: EZ: ezetimibe; H&E: hematoxylin and eosin; NAFLD: non-alcoholic fatty liver disease; NAS: NAFLD activity score; SEM: standard error of mean; TG: triglyceride.

To assess whether ezetimibe is beneficial in hepatic steatosis, we first measured hepatic lipids, including TG, TC, and FFA. Hepatic TG and TC levels were significantly decreased in ezetimibe-treated rat livers, similar to results in plasma (Figures 2A, B). Hepatic FFA levels were not significantly different between groups (Figure 2C). Histological examination of the control liver sections revealed an accumulation of LDs in most hepatocytes, with features of both macrovesicular and microvesicular steatosis, diffuse hepatocyte ballooning, and foci of inflammatory cell infiltrates throughout the lobules, indicating that a high-fat diet resulted in severe steatosis (Figures 2D, E). Compared with the control, ezetimibe treatment significantly reduced lobular inflammation as well as steatosis, leading to a significant decrease in NAFLD activity score (Figures 2D, E). In addition, the number of hepatic LDs decreased after ezetimibe treatment (Figure 2F). Interestingly, ezetimibe showed a decrease in the number of large LDs (>5 µm in diameter) compared with the control (Figure 2G). These results indicate that ezetimibe effectively ameliorated hepatic fat accumulation.

### 3.3 Ezetimibe alters hepatic metabolic profile in an obese and type 2 diabetic rats

**FIGURE 3:**
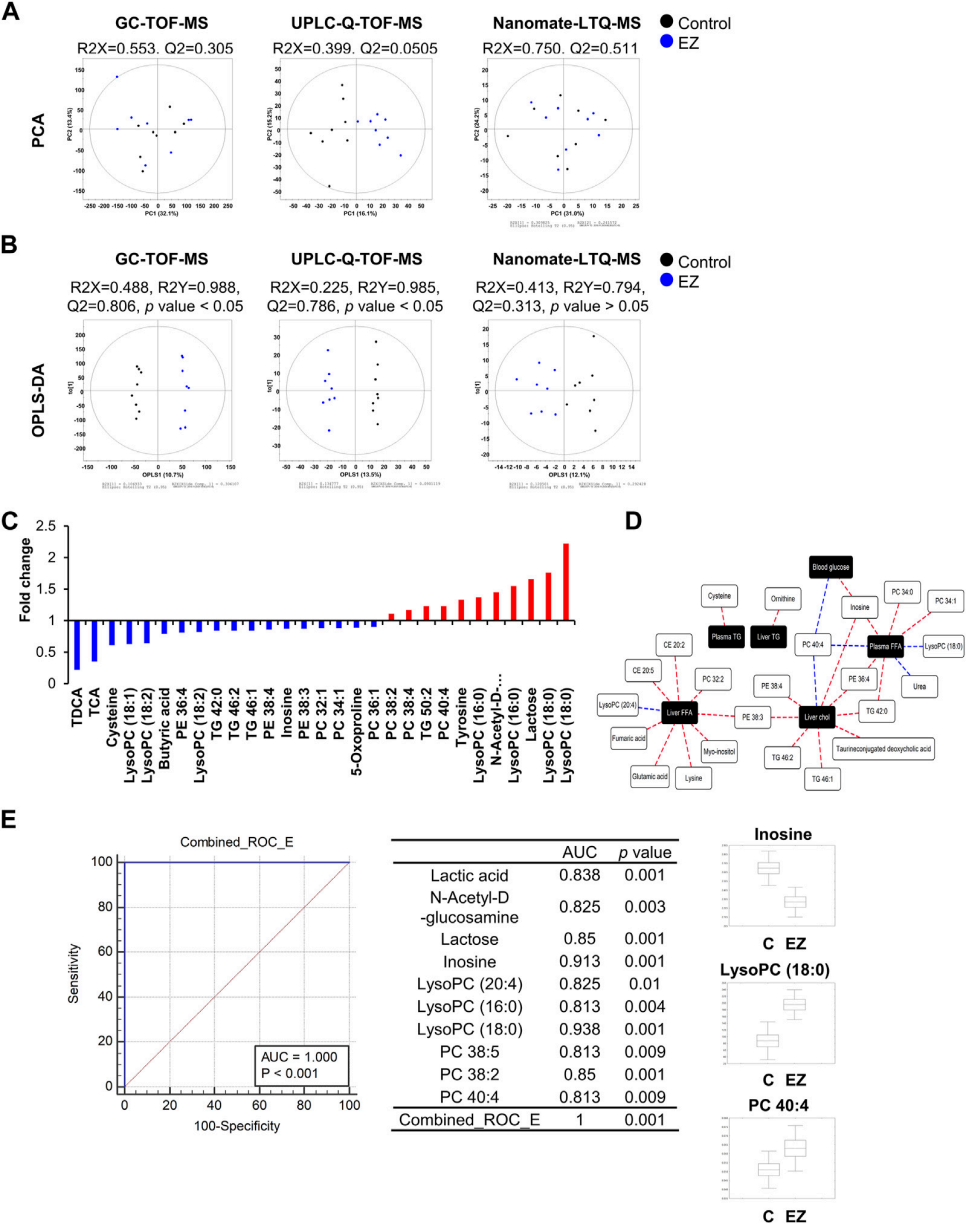
Metabolomic and lipidomic analyses. **(A, B)** Principal component analysis (PCA) **(A)** and orthogonal partial least squares discriminant analysis (OPLS-DA). **(B)** Score plots analyzed by GC-TOF-MS, UPLC-Q-TOF-MS, and Nanomate-LTQ-MS using SIMCA P+ (version 12.0, Umetrics, UMEÅ, Sweden). **(C)** Hepatic metabolites significantly altered (VIP >1.0 and *p*-value <0.05) by ezetimibe treatment from each OPLS-DA model. The significantly different hepatic metabolites were selected based on variable importance projection (VIP) values, and significance was tested by analysis of variance (ANOVA) between experimental groups. The relative level of each metabolite was normalized to that of the control group. **(D)** Correlation network between phenotype data and hepatic metabolites according to Pearson’s correlation coefficient (red line: r > 0.65 or blue line: r < −0.65) in ezetimibe-treated rat livers. **(E)** Combined ROC curves of potential hepatic biomarkers by ezetimibe treatment in OLETF rat livers. Abbreviations: AUC: area under the curve; CONT: control; EZ: ezetimibe; lysoPC: lysophosphatidylcholine; PC: phosphatidylcholine; PE: phosphatidylethanolamine; ROC: receiver operating characteristic; TCA: taurine-conjugated cholic acid; TDCA: taurine-conjugated deoxycholic acid.

Next, we performed global metabolite profiling in rat livers by metabolomic and lipidomic approaches. In the PCA score plot, the GC-TOF-MS and nanomate-LTQ data sets showed that experimental groups were intermingled with each other, but the UPLC-Q-TOF-MS data set was slightly clustered by each group (Figure 3A). However, as shown in Figure 3B, OPLS-DA score plots derived from GC-TOF-MS, UPLC-Q-TOF-MS, and nanomate-LTQ-MS data sets exhibited clusters according to each experimental group. Through comprehensive metabolite profiling, a total of 129 metabolites were identified (Supplementary Tables S2–S4).

For the selection of hepatic metabolites, cut-off VIP values (>1.0) derived from the OPLS-DA model were used. Relative levels of hepatic metabolites were converted into fold-change, which were normalized by levels in the control group, and visualized in a graph (Figure 3C). Ezetimibe treatment resulted in a significant reduction in levels of 18 metabolites including amino acids (cysteine, 5-oxoproline), fatty acids (butyric acid), bile acids (taurine-conjugated deoxycholic acid (TDCA), taurine-conjugated cholic acid), inosine, lysophosphatidylcholines (lysoPCs) (lysoPC 18:1, lysoPC 18:2), phosphatidylethanolamines (PEs) (PE 36:4, PE 38:4, PE 38:3), phosphatidylcholines (PCs) (PC 32:1, PC 34:1, PC 36:1) and TGs (TG 42:0, TG 46:1) and an increase in 11 metabolites (amino acid (tyrosine), carbohydrates (N-acetyl-D-glucosamine, lactose), lysoPCs (lysoPC 16:0, lysoPC 18:0), PCs (PC 38:2, PC 38:4, PC 40:4), and TG 50:2 (Figure 3C).

We then analyzed the correlation between significantly altered hepatic metabolites and metabolic parameters as important indicators of obesity and T2D risk factors. The correlation network (Figure 3D) was constructed by Pearson’s correlation coefficients (r > 0.65 or r < −0.65). According to ezetimibe treatment (Figure 3D), various lipid-related metabolites (cholesterol ester (CE) 20:2, CE 20:5, PC 32:2, PC 34:0, PC 34:1, PC 40:4, PE 36:4, PE 38:3, TG 42:0, lysoPCs with C18:0 and 20:4) and some organic acids (glutamic acid, fumaric acid, and urea) were highly correlated with liver and plasma free fatty acids. In addition, liver TC showed a positive correlation with PE 38:3, PE 38:4, TG 42:0, TG 46:1, TG 46:2, and TDCA while PC 40:4 showed a negative correlation with ezetimibe treatment in the obese T2D rat model (Figure 3D). We further investigated the discrimination between control and ezetimibe treatment using the area under the receiver operating characteristic (ROC) curve (AUC) (Figure 3E), which is considered a statistically valid and objective means of determining the clinical utility of a biomarker (Klein and Shearer, 2016). The results showed that ten hepatic metabolites, including six lipid metabolites [lysoPC (20:4), lysoPC (16:0), lysoPC (18:0), PC 38:5, PC 38:2, and PC 40:4], two carbohydrates (N-acetyl-D-glucosamine and lactose), lactic acid, and inosine, showed good AUC values (>0.8) by ezetimibe treatment in OLETF rat livers (Figure 3E). Among the metabolites, three hepatic metabolites, inosine, lysoPC (18:0), and PC 40:4, were strongly related to ezetimibe treatment (Figure 3E).

### 3.4 Ezetimibe modulates hepatic phospholipid metabolism to attenuate liver steatosis

Metabolomic and lipidomic profiling revealed significant changes in phospholipids, particularly phosphatidylcholines (PC), accompanied by alterations in their fatty acyl-chain composition. Further analyses indicated that ezetimibe upregulates key genes involved in phospholipid biosynthesis (CTP:phosphocholine cytidylyltransferase alpha and phosphatidylethanolamine N-methyl-transferase) and remodeling (lysophosphatidylcholine acyltransferase 3).

**FIGURE 4:**
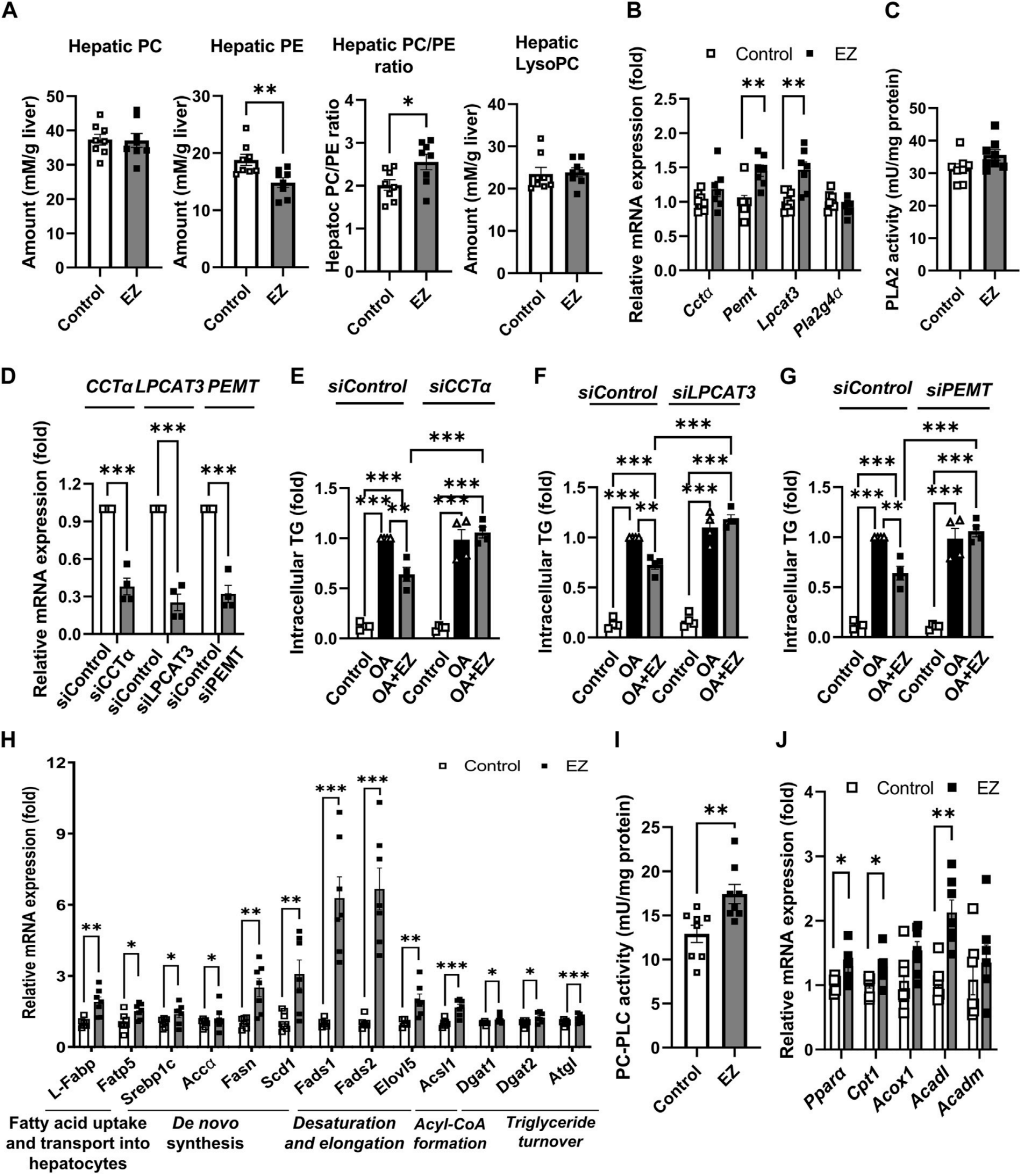
Effect of ezetimibe on hepatic phospholipid and lipid metabolism in OLETF rat liver. **(A)** Quantitative analysis of hepatic levels of total PC and PE, the ratio of PC to PE, and total lysoPC in rat liver. **(B)** Relative mRNA expression of genes related to phosphatidylcholine biosynthesis and remodeling in rat liver. **(C)** PLA_2_ enzyme activity in rat liver (*n* = 8 for each group). **(D)** Relative mRNA expression of *CCTα, LPCAT3, or PEMT* in HepG2 cells after siRNA transfection. **(E–G)** Quantitative analysis of intracellular TG amount in siRNA transfected HepG2 cells prior to ezetimibe treatment with or without 2 mM OA for 18 h (*n* = 4 independent experiments). Relative mRNA expression of genes related to **(H)** *de novo* lipogenesis, and **(I)** fatty acid β-oxidation in rat liver (*n* = 7 for each group). **(J)** PC-PLC enzyme activity in rat liver (*n* = 8 for each group). Data are expressed as the mean ± S.E.M. *p < 0.05, **p < 0.01, ***p < 0.001 vs*.* control, by Student’s t-test. Abbreviations: *Acc1α*: acetyl-CoA carboxylase 1α; *Acadl*: acyl-CoA dehydrogenase long chain; *Acadm*: acyl-CoA dehydrogenase medium chain; *Acox1*: acyl-CoA oxidase 1; *Acsl1*: long-chain acyl-coenzyme A synthetase 1; *Cctα:* CTP:phosphpocholine cytidylyltransferase alpha; *Cpt1-α*: carnitine palmitoyltransferase 1α; *Dgat*: diacylglycerol acyltransferase; *Elovl5*: elongation of very long chain fatty acids protein 5; EZ: ezetimibe; *Fads2*: fatty acid desaturase 2; *Fasn*: fatty acid synthase; *Fatp5*: fatty acid transport protein 5; *L-Fabp*: liver fatty acid-binding protein; *Lpcat*: lysophosphatidylcholine acyltransferase; lysoPC: lysophosphatidylcholine; PC: phosphatidylcholine; PE: phosphatidylethanolamine; *Pemt*: phosphatidylethanolamine N-methyl-transferase; *Pla2g4α:* phospholipase A2 Group IVA; PC-PLC: PC-specific phospholipase C; *Ppar-α*: peroxisome proliferator-activated receptor-α; OA: oleic acid; *Scd1*: stearoyl-CoA desaturase-1; *Srebp1c*: sterol regulatory element-binding protein 1c; TG: triglyceride.

Among lipid metabolism–related metabolites and precursors (fatty acids, lysoPCs, lysoPEs, PCs, PEs, and TGs), phospholipids were notably affected in the liver during ezetimibe treatment. Therefore, we further examined whether ezetimibe treatment affected phospholipid metabolism. Given that hepatic PC amount and the PC to PE ratio are inversely correlated with the development and progression of NAFLD (Li et al., 2006), we measured the hepatic levels of total PC and PE. As shown in Figure 4A, total PC levels in the liver were not significantly different between ezetimibe-treated rats and control rats, whereas total PE levels in the liver were significantly reduced in rats treated with ezetimibe compared to the control group (Figure 4A). The hepatic PC/PE ratio was significantly increased in rats treated with ezetimibe (Figure 4A). In the meantime, when we measured the total lysoPC levels in the rat liver, we observed no significant difference between the total lysoPC levels in the livers of both groups (Figure 4A).

Next, we measured the gene expression and enzyme activities related to phospholipid biosynthesis and remodeling. CTP:phosphocholine cytidylyltransferase alpha (CCTα) and phosphatidylethanolamine N-methyl-transferase (PEMT) are crucial enzymes of PC biosynthesis (van der Veen et al., 2017a). Ezetimibe treatment elevated the mRNA expression of *Cctα* and *Pemt* compared with control (Figure 4B). Lysophosphatidylcholine acyltransferase 3 (*Lpcat3*) and cytosolic phospholipase A2 (PLA2, encoded by *Pla2g4a*) are enzymes that catalyze the incorporation and liberation of polyunsaturated fatty acids (PUFAs) such as arachidonic acid (AA, C20:4), respectively, during phospholipid remodeling (Wang and Tontonoz, 2019). As shown in Figure 4B, ezetimibe treatment significantly upregulated *Lpcat3* mRNA expression, whereas no change was observed in hepatic *Pla2g4a* mRNA by ezetimibe treatment. In addition, activity of PLA2 was not changed (Figure 4C).

We further examined whether the regulation of hepatic PC metabolism is involved in the ameliorative effect of ezetimibe on liver steatosis. Ezetimibe significantly attenuated oleic acid (OA)-induced TG accumulation in the control, but the effects were not observed in HepG2 cells transfected with *CCTα*, *LPCAT3*, or *PEMT* siRNA (Figures 4D–G).

### 3.5 Ezetimibe induces a shift in hepatic lipid metabolism towards long-chain and PUFA-Containing species

As shown in Figure 3C, ezetimibe treatment led to a significant decrease in PCs containing saturated and monounsaturated fatty acids, while elevating levels of PC 38:2, PC 38:4, and PC 40:4—PCs with long-chain PUFAs (Figure 3C). To better understand the effect of ezetimibe on the acyl-chain composition of PCs, we examined gene expressions related to various lipid metabolism aspects including lipid uptake, synthesis, and storage. The mRNA expressions of liver fatty acid-binding protein (*L-Fabp*) and fatty acid transport protein 5 (*Fatp5*), involved in the early steps of long-chain fatty acid uptake/activation, were significantly upregulated in ezetimibe-treated rat livers compared with control livers (Figure 4H). We also observed that ezetimibe elevated gene expression related to *de novo* fatty acid synthesis, including sterol regulatory element-binding protein 1c (*Srebp1c*) and the target genes fatty acid synthase (*Fasn*), acetyl-CoA carboxylase 1α (*Acc1α*), and stearoyl-CoA desaturase-1 (*Scd1*) (Figure 4H). Gene expression of fatty acid desaturase 2 (*Fads2*) and elongase (elongation of very long chain fatty acids protein 5, *Elovl5*), which are required for AA synthesis from dietary linoleic acid, was significantly upregulated in ezetimibe-treated rat livers compared with control livers. Long-chain acyl-coenzyme A synthetases (*Acsl1*), involved in converting fatty acids to acyl-coenzyme A, were also upregulated (Figure 4H). The mRNA expression of DAG acyltransferase (*Dgat*) 1 and 2 and adipose triglyceride lipase (*Atgl*), which encode enzymes that catalyze the synthesis and breakdown of TG, respectively, otherwise known as TG turnover, were upregulated in ezetimibe-treated rat livers compared with the control rat liver (Figure 4H). In addition to mRNA expression, ezetimibe enhanced hepatic activity of PC-specific phospholipase C (PC-PLC), which contributes to TG synthesis from excess PC (van der Veen et al., 2017a), in rat liver compared with control group (Figure 4I). These results indicate a shift in lipid metabolism induced by ezetimibe toward the synthesis of long-chain- and PUFA-containing lipid species. Ezetimibe treatment also promoted fatty acid β-oxidation, thereby reducing fat accumulation in rat livers (Figure 4J). The mRNA levels of peroxisome proliferator-activated receptor-α and its target genes, carnitine palmitoyltransferase 1α, acyl-CoA oxidase 1, and acyl-CoA dehydrogenases, were upregulated in ezetimibe-treated rat livers compared with the control (Figure 4J).

## 4 Discussion

In the current study, we investigated the beneficial effects of ezetimibe on hepatic metabolites and lipids, aiming to elucidate its mechanism in reducing hepatic steatosis. Using OLETF rats on a high-fat diet as a rat model of obesity and T2D, ezetimibe demonstrated improvements in metabolic parameters and reduction in hepatic fat accumulation. The comprehensive metabolomic and lipidomic profiling revealed significant changes in phospholipids, particularly PC, and alterations in the fatty acyl-chain composition in hepatic PCs. Further analyses involving gene expression and triglyceride assessments in rat liver tissues, HepG2 hepatoma cell lines, and siRNA experiments unveiled that ezetimibe’s mechanism involves the upregulation of phospholipid biosynthesis and remodeling.

While several studies have demonstrated the efficacy and molecular mechanisms of ezetimibe on NAFLD in animals and humans (Deushi et al., 2007; Yoneda et al., 2010; Takeshita et al., 2014; Chang et al., 2015b), the exact process by which ezetimibe is beneficial in the treatment of NAFLD remains unclear. In this study, we observed reduced plasma levels of TG, TC, and FFA with ezetimibe treatment. Additionally, hepatic TG and TC levels significantly decreased. Ezetimibe, known for inhibiting intestinal cholesterol absorption, also interacts with hepatic NPC1L1, diminishing biliary cholesterol absorption and lowering serum cholesterol levels (Temel et al., 2007). Indeed, overexpression of hepatic NPC1L1 in the liver leads to free cholesterol accumulation and exacerbation of steatosis (Toyoda et al., 2019). Previous reports have shown high NPC1L1 expression in both the liver and intestine in rats, unlike mice, where NPC1L1 expression is predominantly observed in the intestine and undetectable in the liver (Garcia-Calvo et al., 2005 ), suggesting that ezetimibe’s effects on hepatic steatosis may be attributed, in part, to inhibiting NPC1L1 in the liver, highlighting a potential mechanism for its therapeutic benefits in NAFLD.

In addition to the analysis of conventional lipid parameters, we further identified hepatic metabolites and lipids significantly altered by ezetimibe treatment through metabolomic and lipidomic approaches. Multivariate statistical analysis including correlation analysis and ROC curve analysis is a statistically valid and objective means of determining the clinical utility of a biomarker (Klein and Shearer, 2016). Our results showed that several hepatic metabolites, including inosine, lysoPC (18:0), and PC 40:4, could serve as hepatic biomarker candidates for improvement of NALFD associated with ezetimibe treatment (Figure 3E, Supplementary Figure S1).

**FIGURE 5:**
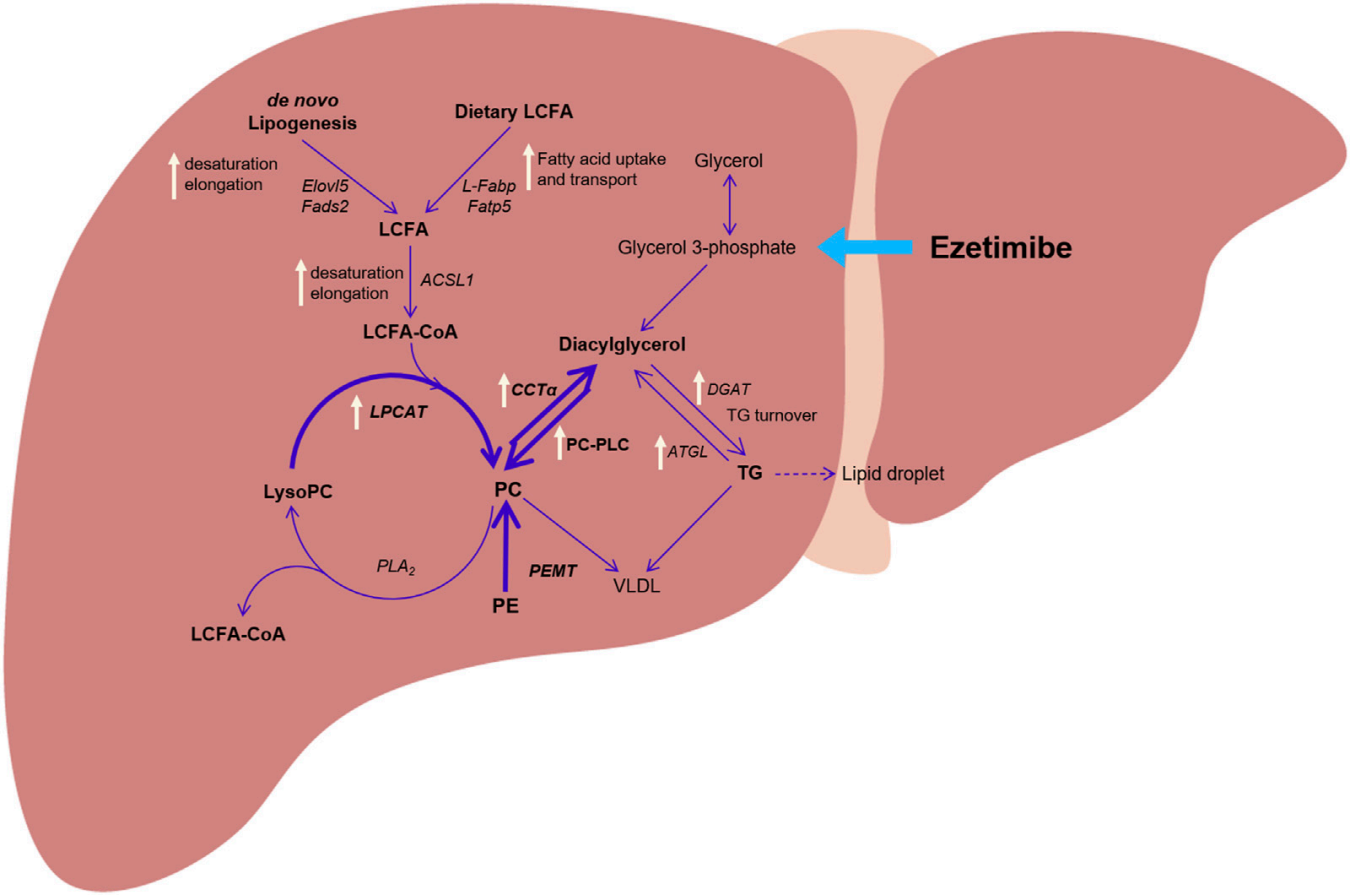
The proposed hepatic metabolic pathway and altered gene expression by treatment with ezetimibe (white arrow) in a rat model of obesity and type 2 diabetes. Abbreviations: *ACSL1*: long-chain acyl-coenzyme A synthetase 1; *CCTα:* CTP:phosphpocholine cytidylyltransferase alpha; *DGAT*: diacylglycerol acyltransferase; *ELOVL5*: elongation of very long chain fatty acids protein 5; *FADS2*: fatty acid desaturase 2; *FATP5*: fatty acid transport protein 5; *L-FABP*: liver fatty acid-binding protein; *LPCAT*: lysophosphatidylcholine acyltransferase; PC: phosphatidylcholine; PE: phosphatidylethanolamine; *PEMT*: phosphatidylethanolamine N-methyl-transferase; PLA_2_ *:* phospholipase A_2_; PC-PLC: PC-specific phospholipase C.

Through comprehensive metabolomic and lipidomic analyses along with analysis of related gene expression, we found significant alterations in phospholipid metabolism and its composition according to ezetimibe treatment (Figure 5). Previous studies have demonstrated that alterations in phospholipid metabolism in the liver are associated with disease progression (Musso et al., 2009). A decrease in the level of hepatic total PC and the ratio of PC to PE was observed in patients with NAFLD and a rodent NAFLD model (Li et al., 2006; Puri et al., 2007; Ling et al., 2012; Arendt et al., 2013; Wattacheril et al., 2013). Li *et al.* demonstrated that a deficiency of liver-specific CCTα or PEMT, which are crucial enzymes of PC biosynthesis, during high-fat diet feeding caused an increase in steatosis and inflammation, accompanied by a decrease in PC/PE ratio, which adversely influenced membrane integrity, leading to liver damage and inflammation (Li et al., 2006). Furthermore, choline supplements reversed the decrease in PC/PE ratio and attenuated liver damage (Li et al., 2006; Ling et al., 2012). We observed that ezetimibe treatment affects phospholipid metabolism in the liver, specifically leading to a decrease in total PE levels and an increase in the PC/PE ratio. Thus, these results suggest that ezetimibe treatment could increase the ratio of PC to PE in the liver, which might be due to the activation of the flux from PE to PC rather than the stimulation of PC synthesis from choline.

Notably, we found that ezetimibe altered the fatty acyl-chain composition in hepatic PCs. Fatty acyl-chain composition in hepatic PCs is linked to NAFLD development, with decreased levels of PCs containing PUFAs like AA and docosahexaenoic acid (DHA, C22:6) in individuals with steatosis and NASH (Puri et al., 2007; Chitraju et al., 2012; Wattacheril et al., 2013). PEMT-mediated PC synthesis and LPCAT3-related PC remodeling produce PC containing PUFAs, such as AA and DHA (DeLong et al., 1999; Wang and Tontonoz, 2019). There are several reports that mice lacking PEMT or with reduced LPCAT3 expression exhibit severe fatty liver conditions (Hashidate-Yoshida et al., 2015; van der Veen et al., 2017b). Conversely, liver-specific overexpression of *Lpcat3* improved plasma lipoprotein metabolic profiles in mice fed a normal chow diet (Cash and Hui, 2016). In addition, ezetimibe did not ameliorate fat accumulation and inflammation in the *Pemt* ^/-^ mouse liver (van der Veen et al., 2017b). Furthermore, the latest research unveiled the pivotal role of LPCAT3 in NASH progression, particularly through its influence on membrane phospholipid composition and mitochondrial homeostasis (Tian et al., 2024). In this study, loss of LPCAT3 increased membrane phospholipid saturation and induced stress-induced autophagy, while liver-specific overexpression of LPCAT3 showed potential in alleviating NASH-related inflammation and fibrosis (Tian et al., 2024). In our study, ezetimibe treatment notably increased *Pemt* and *Lpcat3* mRNA expression in rat livers, along with higher levels of PCs containing PUFA. This suggests that ezetimibe’s protective effects against NAFLD may involve modulation of PC metabolism pathways. Additionally, we observed that knocking down the *CCTα*, *LPCAT3*, or *PEMT* genes in HepG2 cells abolished ezetimibe’s ability to reduce oleate-induced triglyceride accumulation, suggesting the importance of these enzymes in mediating ezetimibe’s efficacy against NAFLD. Therefore, our findings imply the potential of ezetimibe as a therapeutic agent for NAFLD by targeting key enzymes involved in PC biosynthesis and remodeling.

Furthermore, we observed the increased expression of hepatic genes involved in the uptake and activation of long-chain fatty acid (*L-Fabp, Fatp5, Fads2, Elovl5* and *Acsl1*), synthesis of fatty acids (*Srebp1c*, *Fasn*, and *Scd1*) and diacylglycerol (*Lipin1*), and TG turnover (*Dgat* 1 and 2, and *Atgl* by treatment with ezetimibe. L-FABP and FATPs are known to interact with PPARα, leading to regulating long-chain fatty acid metabolism (Frohnert et al., 1999; Hostetler et al., 2009). Our results showed that ezetimibe upregulates the expression of PPARα, influencing long-chain fatty acid metabolism through interactions with *L-Fabp* and *Fatps*. Therefore, these results suggest that ezetimibe might drive lipid metabolism toward the synthesis of long-chain- and PUFA-containing lipid species, including PCs. Additionally, the observed upregulation of PPARα expression in our study may contribute to the modulation of VLDL metabolism, as PPARα is known for its regulatory functions in lipid metabolism, including the metabolism of long-chain fatty acids and its implications in VLDL clearance mechanisms (Deushi et al., 2007; Chan et al., 2010; Engelking et al., 2012). The interaction between PPARα and key genes involved in lipid metabolism, such as L-FABP and FATPs, suggests a potential mechanism through which ezetimibe influences VLDL secretion and clearance (Osipova et al., 2022). These findings provide further insights into the complex effects of ezetimibe on lipid homeostasis, indicating a potential correlation between the observed changes in PPARα expression and alterations in lipid metabolism gene expression following ezetimibe treatment, and suggesting a mechanism through which ezetimibe may affect VLDL clearance and lipid species synthesis, including PCs.

PC amount and composition are reported to influence LD size and number (Guo et al., 2008). Several studies have reported that deficiency of PC related to a choline-deficient diet or knockout of CCTα or PEMT specifically in the liver leads to hepatic TG accumulation full of large LDs, which is a hallmark signature of NAFLD (Jacobs et al., 2004; Li et al., 2006; Guo et al., 2008; Krahmer et al., 2011). LDs are surrounded by a phospholipid monolayer with a core of neutral lipids such as TG (Tauchi-Sato et al., 2002). Insufficient PCs surrounding LD cores result in LD fusion and enlargement (Krahmer et al., 2011). Small LDs are preferentially engulfed by autophagic machinery and degraded within acidic vesicles (Schott et al., 2019). In this study, we observed that, in ezetimibe-treated rat livers, LD number and size decreased, potentially linked to induced autophagy flux reported in previous studies (Yamamura et al., 2014; Chang et al., 2015b). Thus, further investigation is needed to ascertain whether LDs with polyunsaturated fatty acid (PUFA)-containing PC, a result of ezetimibe treatment, undergo degradation or selective autophagy. Understanding these dynamics could shed light on ezetimibe’s effect on LD metabolism and its potential in mitigating NAFLD.

A limitation of this study is the absence of significant changes in blood glucose levels and liver weight following ezetimibe treatment as reported in our previous studies (Chang et al., 2015b). This discrepancy may be explained by different diets and administration duration. The other limitation is that we did not investigate the effect of ezetimibe on lipid metabolism in the intestine and adipose tissue. Several studies showed that ezetimibe has effects on intestinal lipid metabolism, including reducing cholesterol absorption, reducing chylomicron overproduction, and increasing LDL receptor gene expression (Ge et al., 2008; Naples et al., 2012; Drouin-Chartier et al., 2016). There are also reports that ezetimibe may have positive potentials on adipose tissue, including reducing inflammation, regulating hormonal function, and reducing lipid accumulation (Krysiak et al., 2014; Cho et al., 2020). In our previous study, we observed no significant change in epididymal fat mass, but with increasing levels of plasma adiponectin after ezetimibe treatment (Yang et al., 2011; Chang et al., 2015a). Therefore, we cannot exclude the possibility of the effect of ezetimibe on lipid metabolism in adipose tissue and intestine. More research is needed to fully understand the effects of ezetimibe on intestine and adipose tissue and how these effects may impact overall health. Despite this limitation, our findings confirm that ezetimibe can efficiently improve hepatic steatosis as well as plasma lipid profiles, followed by significant changes in hepatic metabolites and lipids, and the gene expression related to lipid metabolism including phospholipids.

## 5 Conclusion

In conclusion, our study demonstrated the effect of ezetimibe on hepatic steatosis through profiling hepatic metabolites and lipids in OLETF rats fed a high-fat diet as a rat model of obesity and T2D and evaluating alteration of gene expression related to phospholipid biosynthesis and remodeling. Our findings suggest that ezetimibe significantly regulates lipid metabolism toward increasing the production of PUFA-containing PC species and decreasing the number and size of LDs. These findings provide valuable insights into the molecular pathways through which ezetimibe mitigates hepatic fat accumulation, emphasizing the role of phospholipid metabolism.

## Data Availability

All relevant data is contained within the article. The original contributions presented in the study are included in the article/Supplementary Material, further inquiries can be directed to the corresponding author/s.
