# Supplementary material for: Ezetimibe, Niemann-Pick C1 like 1 inhibitor, modulates hepatic phospholipid metabolism to alleviate fat accumulation
Source: Front Pharmacol. 2024 Jun 17;15:1406493. doi: 10.3389/fphar.2024.1406493 (PMC11215075; doi:10.3389/fphar.2024.1406493)
Supplement: Supplementary file 1 [file DataSheet1.DOCX]

Supplementary Material

**Ezetimibe, Niemann-Pick C1 like 1 inhibitor, modulates hepatic phospholipid metabolism to alleviate fat accumulation**

Hyekyung Yang^1,§^, Dong Ho Suh^2,§^, Eun Sung Jung^2^, Yoonjin Lee^1^, Kwang-Hyeon Liu^3^, In-Gu Do^4^, Choong Hwan Lee^2,*^, Cheol-Young Park^1,5,*^

^1^Medical Research Institute, Kangbuk Samsung Hospital, Sungkyunkwan University School of Medicine, Seoul 03181, South Korea

^2^Department of Bioscience and Biotechnology, Konkuk University, Seoul 05029, South Korea

^3^College of Pharmacy and Research Institute of Pharmaceutical Sciences, Kyungpook National University, Daegu, 41566, South Korea

^4^Department of Pathology, Kangbuk Samsung Hospital, Sungkyunkwan University School of Medicine, Seoul 03181, South Korea

^5^Division of Endocrinology and Metabolism, Department of Internal Medicine, Kangbuk Samsung Hospital, Sungkyunkwan University School of Medicine, Seoul 03181, South Korea.

**Table of contents**

**Supplementary Figure S1**. Scatter plot analysis between metabolites and liver damage markers

**Supplementary Table S1**. Primer sequences used for real-time polymerase chain reactions.

**Supplementary Table S2**. Identification of hepatic metabolites from GC-TOF-MS data.

**Supplementary Table S3.** Identification of hepatic metabolites from UPLC-Q-TOF-MS data.

**Supplementary** **Table S4**. Identification of hepatic metabolites from Nanomate-LTQ-MS data.


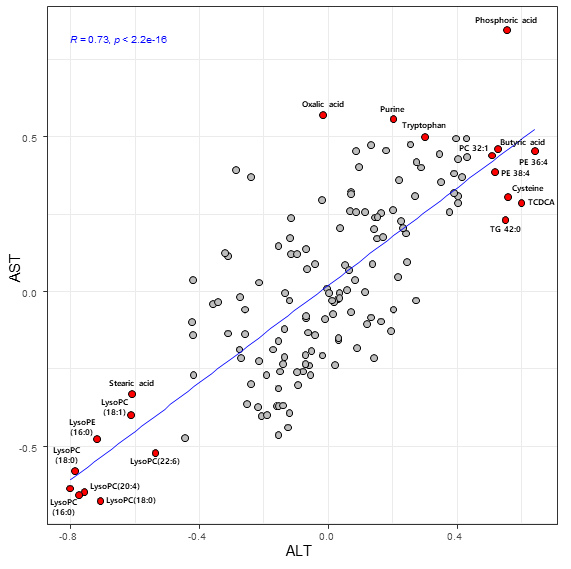


**Supplementary Figure S1. Scatter plot analysis between metabolites and liver damage markers**

**Supplementary Table S1. Primer sequences used for real-time polymerase chain reactions.**

| Gene symbol | Forward | Reverse |
| --- | --- | --- |
| *Rat* |  |  |
| *Cctα* | GCCAGCTCCTTTTTCTGATG | TCCATCCGCATAAACTCTCA |
| *Pemt* | TCTGGAATGTGGTAGCAAGGT | TAGGTAAGGGGACCCGAAGG |
| *Lpcat3* | CAGACCATCCACTGGCTTTT | TTGGCACCATTGCTTTGTAA |
| *Pla2g4a* | GCACATAATAGTGGAACACC | CTTGTTCGCTTCCTGCTGTC |
| *L-Fabp* | GGTCCAAGGTGATCCACAAT | CCCTCCATCTTAACCACTGC |
| *Fatp5* | TCCTCGGCTGCTTACAACTT | TTCACCCACATACAGGACCA |
| *Fads2* | CAAGGACCCCGACATAAAGA | CTGGTGGTTGTAGGGCAGAT |
| *Srebp1c* | GTACTCAAGCCTGCCTTCC | TCTGGACCTGGGTGTGTAAG |
| *Fasn* | TTCAGGGAACGGGTATTGCC | AATGTCACGCCTTGCTCCTT |
| *Acc1α* | CTGTGAGGTGGATCAGAGAT | TTCAGCTCTAACTGGAAAGC |
| *Scd1* | AGCTGGTGATGTTCCAGAGG | CAAGAAGGTGCTGACGAACA |
| *Fads1* | GGAAGAAATGGGTGGACTTG | CACAAACCAGTTGCTCTCCA |
| *Fads2* | CAAGGACCCCGACATAAAGA | CTGGTGGTTGTAGGGCAGAT |
| *Elovl5* | GAGGCATCCTGGTGGTGTAT | CGTGTTCCCTGACAGAAGAAG |
| *Acsl1* | TTACACACGGGGGACATTG | TCCTGTCGATAATCTTCAAGGTG |
| *Dgat*1 | TCTCTTAAAGCTGGCGGTCC | CCAGTCCCTGTAGAACTCGC |
| *Dgat*2 | CCGCAGCGAGAACAAGAAT | TTGAGCCAGGTGACAGAGAA |
| *Atgl* | GGATCGGATTCTAGAGCACC | GTCTTTCGGTTCCACACAGG |
| *Pparα* | TGCGGACTACCAGTACTTAGGG | GCTGGAGAGAGGGTGTCTGT |
| *Cpt1* | GCACGAGGGAAAAATAAGCA | TCAAACCACCTGTCAAAGCA |
| *Acox1* | CACCTTCGAGGGAGAGAACA | CGCACCTGGTCGTAGATTTT |
| *Acadl* | AGCTCCCACAGGAAAGGCTT | CTCGAGCATCCACGTAGGCT |
| *Acadm* | CTTAGCTTCTGCCCTGTGGT | CAAAATGAGAGACACTTCTCAGGA |
| *Actb* | CTCTTCCAGCCTTCCTTCCT | CGGATGTCAACGTCACACTT |
| *Human* |  |  |
| *CCTα* | GAAGAAGCCAGCAGAGGAAC | GCGTGACCAGAGTGAAATAAGT |
| *PEMT* | CTGGAATGTGGTTGCACGATG | AGCAGGATGGTGACGCTTA |
| *LPCAT3* | CCGTCCTCACTACCTTTTGC | TGATCTTTCCCTCCGTCAAA |

**Supplementary Table S2.** Identification of hepatic metabolites from GC-TOF-MS data.

| **RT^a^** | **Metabolites** | **TMS^b^** | **Unique mass** | **Mass fragments** | **Fold change**  **(EZ/CONT)** |
| --- | --- | --- | --- | --- | --- |
|  |  |  |  |  |  |
| 4.87 | Pyruvic acid | 1TMS | 174 | 59,73,89,99,115,158,174,189 | 1.16 |
| 4.99 | Lactic acid | 2TMS | 117 | 73,117,147,191 | 0.83 |
| 7.84 | Fumaric acid | 2TMS | 245 | 73,147,217,245 | 0.94 |
| 9.14 | Malic acid | 3TMS | 233 | 55,73,101,117,133,147,189,217,233,265,307,335 | 1.08 |
| 9.83 | Glutaric acid | 3TMS | 129 | 73,129,147,231,247,349 | 1.44 |
| 10.63 | Taurine | 3TMS | 326 | 59,73,86,100,147,174,225,248,326 | 0.46 |
| 7.53 | Glycine | 3TMS | 174 | 59,73,86,100,117,133,147,158,174,248,276 | 0.87 |
| 8.02 | Serine | 3TMS | 204 | 59,73,100,116,133,147,174,188,204,218,278 | 1.02 |
| 8.27 | Threonine | 3TMS | 117 | 57,73,86,101,117,147,203,219,291 | 1.24 |
| 9.46 | 5-Oxoproline* | 2TMS | 156 | 59,73,84,100,133,147,156,230,258 | 0.89 |
| 9.70 | Cysteine* | 3TMS | 220 | 59,73,100,116,132,147,163,204,220,294 | 0.57 |
| 10.19 | Glutamic acid | 3TMS | 128 | 56,73,84,100,114,128,147,204,230,246,258,348 | 0.97 |
| 11.90 | Lysine | 4TMS | 174 | 59,73,86,100,156,174,200 | 1.02 |
| 12.51 | Tyrosine* | 4TMS | 218 | 59,73,100,117,133,147,179,218,280 | 1.33 |
| 14.24 | Tryptophan | 2TMS | 202 | 73,202,348 | 0.92 |
| 6.04 | Butyric acid* | 2TMS | 117 | 73,117,147,191,233 | 0.76 |
| 13.08 | Palmitic acid | 1TMS | 117 | 73,117,129,185,313 | 1.05 |
| 14.13 | Linoleic acid | 1TMS | 117 | 67,103,117,129,147,262,337 | 1.26 |
| 14.27 | Stearic acid | 1TMS | 117 | 55,73,117,129,145,185,201,341 | 1.23 |
| 19.64 | Cholesterol | 1TMS | 129 | 73,129,213,255,329,368,458 | 1.30 |
| 7.21 | Glycerol | 3TMS | 103 | 59,73,89,103,117,133,147,205,218 | 0.85 |
| 12.34 | Glucose | 5TMS | 205 | 59,73,89,103,117,147,189,205,229,319 | 0.95 |
| 12.45 | Glucose | 5TMS | 205 | 59,73,89,103,129,147,160,205,319 | 0.92 |
| 12.58 | Adonitol | 5TMS | 103 | 59,73,89,103,117,129,147,189,217,319 | 1.24 |
| 13.50 | N-Acetyl-D-glucosamine* | 4TMS | 103 | 59,73,87,103,129,147,173,189,274,319,333 | 1.50 |
| 16.82 | Lactose* | 8TMS | 204 | 59,73,103,129,147,169,191,204,217,243,271,305,331,361 | 1.72 |
| 17.17 | Maltose | 8TMS | 204 | 59,73,103,129,147,169,191,204,217,243,271,305,331,361 | 1.10 |
| 13.07 | Purine | 3TMS | 353 | 59,73,84,100,147,238,294,353,368 | 1.07 |
| 15.60 | Uridine | 3TMS | 217 | 73,103,147,169,191,217,259,299,445 | 0.94 |
| 16.22 | Inosine* | 4TMS | 217 | 59,73,103,129,147,169,193,217,245,281 | 0.86 |
| 6.94 | Benzoic acid | 1TMS | 105 | 51,77,105,135,179,194 | 0.92 |
| 11.62 | Hypoxanthine | 2TMS | 265 | 58,73,84,100,125,147,193,206,2328,265,280 | 1.21 |
| 12.81 | Shikimic acid | 4TMS | 204 | 59,73,103,147,167,189,204 | 0.94 |

| ^a^RT, retention time; ^b^TMS, trimethylsilyl; |
| --- |
| *Metabolites showing significant differences (VIP > 1.0 and *p* value < 0.05) between experimental groups |

**Supplementary Table S3.** Identification of hepatic metabolites from UPLC-Q-TOF-MS data.

| **RT^a^** | **Mass** | **Metabolites** | **MW** | **HMDB^b^ formula** | **Fold change**  **(EZ/CONT)** |
| --- | --- | --- | --- | --- | --- |
| 6.30 | 514.2866 | Taurine‐conjugated cholic acid* | 515 | C26H45NO7S | 0.91 |
| 7.69 | 498.2887 | Taurine‐conjugated deoxycholic acid* | 499 | C26H45NO6S | 0.42 |
| 8.14 | 524.2799 | LysoPE (22:6) | 525 | C27H44NO7P | 0.56 |
| 8.16 | 552.3109 | LysoPC (22:6) | 567 | C30H50NO7P | 0.77 |
| 8.19 | 504.3123 | LysoPC (18:2)* | 519 | C26H50NO7P | 0.65 |
| 8.26 | 524.2782 | LysoPE (22:6) | 525 | C27H44NO7P | 0.78 |
| 8.29 | 552.309 | LysoPC (22:6) | 567 | C30H50NO7P | 1.12 |
| 8.34 | 500.2786 | LysoPE (20:4) | 501 | C25H44NO7P | 1.02 |
| 8.36 | 528.3109 | LysoPC (20:4) | 543 | C28H50NO7P | 1.16 |
| 8.37 | 504.3113 | LysoPC (18:2)* | 519 | C26H50NO7P | 0.83 |
| 8.60 | 480.3122 | LysoPC (16:0)* | 495 | C24H50NO7P | 1.56 |
| 8.79 | 452.2777 | LysoPE (16:0) | 453 | C21H44NO7P | 1.46 |
| 8.80 | 480.3087 | LysoPC (16:0)* | 495 | C24H50NO7P | 1.38 |
| 8.85 | 506.3282 | LysoPC (18:1)* | 521 | C26H52NO7P | 0.64 |
| 9.03 | 506.3272 | LysoPC (18:1) | 521 | C26H52NO7P | 1.16 |
| 9.65 | 508.3431 | LysoPC (18:0)* | 523 | C26H54NO7P | 2.25 |
| 9.86 | 508.3390 | LysoPC (18:0)* | 523 | C26H55NO7P | 1.78 |

| ^a^RT, retention time; ^b^HMDB, The Human Metabolome Database (www.hmdb.ca); MW, molecular weight. |
| --- |
| *Metabolites showing significant differences (VIP > 1.0 and *p* value < 0.05) between experimental groups |
| Two forms of lysoPC and lysoPE with a fatty acyl chain at sn-1 and sn-2 were detected. |

**Supplementary Table S4.** Identification of hepatic metabolites from Nanomate-LTQ-MS data.

| **m/z** | **Metabolites** | **Adduct** | **Fold change**  **(EZ/CONT)** |
| --- | --- | --- | --- |
| 610.3 | DG 34:2 | NH4+ | 0.89 |
| 612.4 | DG 34:1 | NH4+ | 0.95 |
| 640.6 | DG 36:1 | NH4+ | 1.05 |
| 664.6 | CE 18:3 | NH4+ | 0.93 |
| 688.5 | CE 20:5 | NH4+ | 0.88 |
| 690.5 | CE 20:4 | NH4+ | 0.94 |
| 692.6 | CE 20:3 | NH4+ | 1.19 |
| 694.5 | CE 20:2 | NH4+ | 1.16 |
| 740.5 | TG 42:0* | NH4+ | 0.84 |
| 752.6 | PC 32:2 | Na+ | 0.99 |
| 754.6 | PC 32:1* | Na+ | 0.88 |
| 762.8 | PE 36:4* | Na+ | 0.81 |
| 764.5 | PE 38:6 | H+ | 0.93 |
| 766.6 | PE 38:5 | H+ | 1.00 |
| 768.6 | PE 38:4* | H+ | 0.86 |
| 770.6 | PE 38:3* | H+ | 0.87 |
| 778.6 | PC 34:3 | Na+ | 1.02 |
| 780.7 | PC 34:2 | Na+ | 0.95 |
| 782.7 | PC 34:1* | Na+ | 0.88 |
| 784.8 | PC 34:0 | Na+ | 0.89 |
| 786.7 | PC 36:2 | H+ | 0.94 |
| 788.8 | PC 36:1* | H+ | 0.90 |
| 790.7 | PC 36:0 | H+ | 1.08 |
| 792.6 | TG 46:2* | NH4+ | 0.84 |
| 794.7 | TG 46:1* | NH4+ | 1.09 |
| 796.7 | TG 46:0 | NH4+ | 1.05 |
| 798.7 | PC 35:0 | Na+ | 0.93 |
| 802.7 | PC 36:5 | Na+ | 1.02 |
| 804.7 | PC 36:4 | Na+ | 1.11 |
| 806.7 | PC 36:3 | Na+ | 1.02 |
| 808.7 | PC 38:5 | H+ | 1.17 |
| 810.7 | PC 38:4* | H+ | 1.09 |
| 812.8 | PC 38:3 | H+ | 1.11 |
| 814.8 | PC 38:2* | H+ | 1.11 |
| 818.7 | TG 48:3 | NH4+ | 1.04 |
| 820.6 | TG 48:2 | NH4+ | 0.98 |
| 822.7 | TG 48:1 | NH4+ | 1.09 |
| 824.7 | TG 48:0 | NH4+ | 1.05 |
| 826.7 | PC 38:7 | Na+ | 1.10 |
| 828.7 | PC 38:6 | Na+ | 1.27 |
| 834.7 | TG 49:2 | NH4+ | 1.06 |
| 836.7 | TG 49:1 | NH4+ | 1.07 |
| 838.8 | TG 49:0 | NH4+ | 1.03 |
| 842.7 | PC 40:2 | H+ | 1.06 |
| 844.6 | TG 50:4 | NH4+ | 1.09 |
| 846.7 | TG 50:3 | NH4+ | 1.20 |
| 848.7 | TG 50:2* | NH4+ | 1.23 |
| 850.7 | TG 50:1 | NH4+ | 1.26 |
| 852.8 | TG 50:0 | NH4+ | 1.23 |
| 860.7 | PC 40:4* | Na+ | 1.23 |
| 864.7 | TG 51:1 | NH4+ | 1.12 |
| 870.7 | TG 52:5 | NH4+ | 1.08 |
| 872.7 | TG 52:4 | NH4+ | 1.05 |
| 874.7 | TG 52:3 | NH4+ | 1.11 |
| 876.7 | TG 52:2 | NH4+ | 1.14 |
| 878.8 | TG 52:1 | NH4+ | 1.13 |
| 880.8 | TG 52:0 | NH4+ | 1.14 |
| 886.6 | TG 53:4 | NH4+ | 1.01 |
| 888.7 | TG 53:3 | NH4+ | 1.07 |
| 890.7 | TG 53:2 | NH4+ | 1.21 |
| 892.7 | TG 53:1 | NH4+ | 1.09 |
| 896.7 | TG 54:6 | NH4+ | 1.02 |
| 898.7 | TG 54:5 | NH4+ | 1.02 |
| 900.7 | TG 54:4 | NH4+ | 1.05 |
| 902.7 | TG 54:3 | NH4+ | 1.10 |
| 912.7 | TG 55:5 | NH4+ | 1.09 |
| 914.7 | TG 55:4 | NH4+ | 1.21 |
| 916.7 | TG 55:3 | NH4+ | 1.03 |
| 918.7 | TG 55:2 | NH4+ | 0.94 |
| 920.7 | TG 55:1 | NH4+ | 0.94 |
| 922.7 | TG 55:0 | NH4+ | 0.99 |
| 924.7 | TG 56:6 | NH4+ | 1.07 |
| 928.8 | TG 56:4 | NH4+ | 1.17 |
| 930.7 | TG 56:3 | NH4+ | 1.02 |
| 934.7 | TG 56:1 | NH4+ | 0.97 |
| 942.7 | TG 57:4 | NH4+ | 0.93 |
| 944.6 | TG 57:3 | NH4+ | 0.88 |
| 946.7 | TG 57:2 | NH4+ | 0.89 |
| 952.7 | TG 58:6 | NH4+ | 1.08 |
| 954.8 | TG 58:5 | NH4+ | 1.04 |

*Metabolites showing significant differences (VIP > 1.0 and *p* value < 0.05) between experimental groups
